# An interRAI derived frailty index predicts acute hospitalizations in older adults residing in retirement villages: A prospective cohort study

**DOI:** 10.1371/journal.pone.0264715

**Published:** 2022-03-02

**Authors:** Katherine Bloomfield, Zhenqiang Wu, Annie Tatton, Cheryl Calvert, Nancye Peel, Ruth Hubbard, Hamish Jamieson, Joanna Hikaka, Michal Boyd, Dale Bramley, Martin J. Connolly

**Affiliations:** 1 Department of Medicine, Faculty of Medicine and Health Sciences, University of Auckland, Auckland, New Zealand; 2 Waitematā District Health Board, Auckland, New Zealand; 3 Auckland District Health Board, Auckland, New Zealand; 4 Centre for Health Services Research, University of Queensland, Brisbane, Queensland, Australia; 5 Department of Medicine, University of Otago, Christchurch, New Zealand; University of Malaya, MALAYSIA

## Abstract

**Objectives:**

The development of frailty tools from electronically recorded healthcare data allows frailty assessments to be routinely generated, potentially beneficial for individuals and healthcare providers. We wished to assess the predictive validity of a frailty index (FI) derived from interRAI Community Health Assessment (CHA) for outcomes in older adults residing in retirement villages (RVs), elsewhere called continuing care retirement communities.

**Design:**

Prospective cohort study.

**Setting and participants:**

34 RVs across two district health boards in Auckland, Aotearoa New Zealand (NZ). 577 participants, mean age 81 years; 419 (73%) female; 410 (71%) NZ European, 147 (25%) other European, 8 Asian (1%), 7 Māori (1%), 1 Pasifika (<1%), 4 other (<1%).

**Methods:**

interRAI-CHA FI tool was used to stratify participants into fit (0–0.12), mild (>0.12–0.24), moderate (>0.24–0.36) and severe (>0.36) frail groups at baseline (the latter two grouped due to low numbers of severely frail). Primary outcome was acute hospitalization; secondary outcomes included long-term care (LTC) entry and mortality. The relationship between frailty and outcomes were explored with multivariable Cox regression, estimating hazard ratios (HRs) and 95% confidence intervals (95%CIs).

**Results:**

Over mean follow-up of 2.5 years, 33% (69/209) of fit, 58% (152/260) mildly frail and 79% (85/108) moderate-severely frail participants at baseline had at least one acute hospitalization. Compared to the fit group, significantly increased risk of acute hospitalization were identified in mildly frail (adjusted HR = 1.88, 95%CI = 1.41–2.51, p<0.001) and moderate-severely frail (adjusted HR = 3.52, 95%CI = 2.53–4.90, p<0.001) groups. Similar increased risk in moderate-severely frail participants was seen in LTC entry (adjusted HR = 5.60 95%CI = 2.47–12.72, p<0.001) and mortality (adjusted HR = 5.06, 95%CI = 1.71–15.02, p = 0.003).

**Conclusions and implications:**

The FI derived from interRAI-CHA has robust predictive validity for acute hospitalization, LTC entry and mortality. This adds to the growing literature of use of interRAI tools in this way and may assist healthcare providers with rapid identification of frailty.

## Introduction

The number of retirement villages (RVs), known elsewhere as continuing care retirement communities (CCRCs), has grown significantly over recent years in Aotearoa New Zealand (NZ) [1]. While few RVs existed in the 1980s, approximately 14% of those aged 75 years or older were living in RVs in 2019 [1]. RVs offer a range of facilities and supports, varying from stand-alone secure units, to home/personal care and healthcare supports, with many in NZ also having long-term residential care (LTC) amenities, such as nursing home/private hospital, dementia and psychogeriatric care, on-site. The perceived availability of health and care supports is one of several deciding factors for relocation to RVs identified in those financially able to choose RV lifestyles [2, 3]. Our earlier research suggests many residing in RVs live with considerable unmet health needs and symptoms [2, 4], and may represent those with greater dependency than their community-dwelling peers. Consistent with this, some frail older adults perceive RV living as an alternative to LTC [5].

The interRAI suite of tools assesses health, functional and social needs in different settings [6, 7], and in NZ has been mandated for use in community-dwelling older adults requiring government subsidised home and personal care support, and those in LTC. The interRAI-Community Health Assessment (CHA) is one of several interRAI tools designed to assess health, functional and social needs in community-dwelling older adults [8]. We recently developed a frailty index (FI) from interRAI-CHA and found it was associated with healthcare utilization in the 12 months *prior to* assessment in older adults residing in RVs [9]. Here we report the ability of this FI tool to *prospectively* predict healthcare outcomes (acute hospitalization, LTC entry and mortality).

## Methods

This report was based on a cohort from the “Older People in Retirement Village” study—a study of health, functional and social needs of older adults living in RVs in the Auckland and Waitematā District Health Board (ADHB/WDHB) areas in Auckland, NZ. The trial was registered with the Australia and New Zealand Clinical Trials Registry (ACTRN12616000685415). Ethical approval was obtained from NZ Health and Disability Ethics Committee (16/CEN/34AM05). Detailed methods are reported elsewhere [10], and are briefly reviewed here. The current study is reported according to STROBE guidelines [11].

Settings/participants: All 65 RVs in ADHB and WDHB from July 2016 to June 2018 were eligible for inclusion. Recruitment occurred through gerontology nurse specialist (GNS) contact with RV managers. After recruitment of RVs, residents were individually approached by GNSs. We originally planned to recruit all participants by random sampling methods in larger RVs (those with ≥60 units/apartments) and by approaching all residents in small RVs. However, due to issues accessing some RVs, detailed elsewhere [12], this became impractical and volunteer participants were also sought. Volunteers were recruited by RV newsletters, posters and resident meetings. Sampling was permitted in 17 RVs with residents approached by letter and door knock. All residents in selected units were invited to participate. Residents were excluded if they refused or did not have capacity to consent due to significantly impaired cognitive function (Addenbrook Cognitive Assessment Revised <65) [13], or were thought to possibly lack capacity by GNS, RV manager or general practitioner, in keeping with NZ ethical and legislative requirements. All participants gave written informed consent.

Variables, data sources/measurement: interRAI-CHA assessment was facilitated by trained GNSs unless an interRAI-Home Care (HC) had been performed within the last six months, in which case this was used. InterRAI consists of a series of tools with detailed items addressing health, function, social and psychological domains based on comprehensive geriatric assessment. The interRAI CHA, Contact Assessment (CA) and HC tools are designed for community use [6–8] The CHA is abbreviated in comparison to the HC, however contains embedded screening questions that trigger a Functional Supplement (FS) [8] that when completed, make it comparable to the HC. Additionally, a custom-designed study questionnaire was completed detailing health, function and social aspects of life, including those specifically related to RV living, such as decision-making factors in choosing to live in RV and utilisation of facilities. This information was not used in FI construction. Participants at risk of functional decline or with unmet health needs were identified [10, 14, 15] and enrolled in a randomised controlled trial (RCT) of a multi-disciplinary team (MDT) intervention versus usual care.

InterRAI-CHA items were used to generate an FI based on the cumulative deficit model [9]. The FI had 57 variables, with most scored in a binary manner (absent 0, present 1), and ordinal or continuous variables scored between 0 and 1 (e.g. 0, 0.5, 1). The number of deficits present were summed and divided by the total number of deficits included in the FI model, in accordance with individual scoring in FI models [16, 17]. In those with interRAI-HC, items of commonality between the tools were used for FI construction, with the denominator reduced accordingly for items missing from the HC. As RV residents are considered to be community dwelling, and early results demonstrated similar mean and distribution to FI utilisation in community-dwelling older adults in the UK [18], the following frailty groups were identified: fit (FI 0–0.12), mildly frail (>0.12–0.24), moderately frail (>0.24–0.36) and severely frail (>0.36). Due to small numbers of severely frail, moderate and severely frail groups were combined.

Outcomes included acute hospitalizations, LTC entry and mortality from the date of interRAI-CHA completion during baseline assessment. This information was provided by the Ministry of Health (MoH), matched by unique, encrypted National Health Index identifiers used in the NZ health system. LTC entry was defined as date LTC stay started as per interRAI Long Term Care Facility assessment (mandated for NZ LTC admissions), or earliest date of the means-tested aged residential care government-funded subsidy payment. NZ deprivation index [19] was also sourced from MoH, as an estimation of socioeconomic deprivation based on nine variables obtained at census.

Bias: Recruitment occurred by both sampling and volunteers due to restricted access to some RVs, which impacts on generalisabilty of results [10, 12]. Recruitment of volunteers was by RV newsletters, meetings, posters and word-of-mouth. No investigators were involved in outcome data collection.

Sample size: Calculation of sample size was determined as per need for power of the RCT phase of study, estimating that 572 residents would be required in the initial phase (detailed elsewhere) [10].

Quantitative variables and statistical analyses: Categorical and continuous variables are presented as n (%) and mean (standard deviation, SD), respectively. One way analysis of variance (ANOVA) was used to examine differences in mean frailty index by characteristics of residents. The non-parametric Kaplan-Meier method was used to estimate time to first acute hospitalization, LTC and mortality. The difference in time to the first event by frailty groups was assessed by log-rank test. Multivariable Cox proportional hazards regression models with robust sandwich variance estimates were performed with hazard ratios (HRs) and 95% confidence intervals (CI), to explore the relationship of FI groups with first acute hospitalization, LTC entry and mortality at follow-up. For participants with more than one acute hospitalizations after baseline assessment, the first acute hospitalization was used to calculate the time to event. Participants without a record of hospitalization, LTC admission or mortality were censored at date of death (if died) or otherwise at the end of the follow up period. Four participants were receiving LTC (within the RV) at baseline and were excluded from LTC entry outcome. Multivariable regression was adjusted for age, gender, ethnicity, marital status, NZ deprivation index (as a marker of socioeconomic status), history of acute hospitalizations in 1-year prior to interRAI assessment and MDT intervention (as a time-dependent variable) [20–22]. Overall performance (explained variation) of FI itself for predicting healthcare outcomes was assessed using Nagelkerke’s R^2^, and the discriminative ability was indicated by the Harrell’s concordance (c) statistic [23]. All analyses were performed with SAS v9.4 software (SAS Institute Inc., Cary, NC, USA). A two-sided p <0.05 was considered statistically significant.

## Results

**Fig 1:**
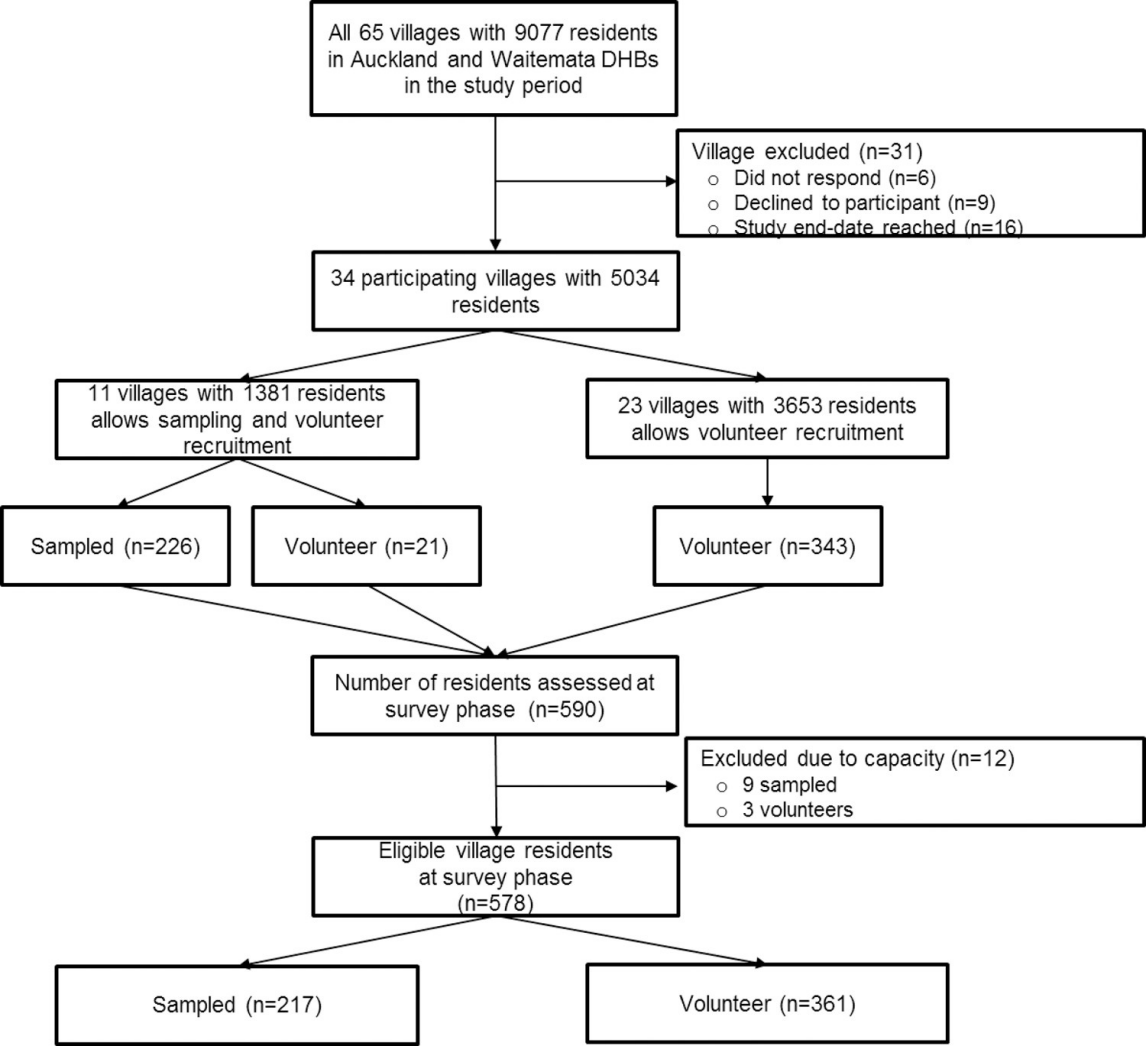
Flow diagram of recruitment of RVs and eligible residents.

Fig 1 shows a flow diagram of eligible residents and recruitment.

Five-hundred-and-seventy-eight subjects (361 volunteers from 296 units in 27 RVs, 217 sampled from 190 sampled units in 11 RVs; 6 RVs allowed both recruitment methods) were recruited from 34 RVs (5034 residents) in the Auckland and Waitematā DHB regions from July 2016 to June 2018. The response rate in randomly sampled units was 35%. Twelve residents were excluded as considering lacking capacity to give informed consent (9 sampled, 3 volunteers). interRAI- derived FI data was available for 577 (FI items obtained from interRAI-HC in 12 subjects). Baseline characteristics and frailty have been reported elsewhere [9] and summarised in Table 1. Mean (SD) age was 81 (7) years, 419 (73%) were female, and 557 (97%) were European, 8 (1%) Asian, 7 (1%) Māori, 1 (<1%) Pasifika, and 4 other (<1%). Table 1 illustrates the mean frailty index across sociodemographic characteristics.

**Table 1:** Mean frailty index by baseline sociodemographic characteristics.

Variable	Number of residents (%)	Frailty index mean (SD)	P value for frailty index difference
Overall	577 (100)	0.16 (0.09)	NA
Gender			0.32
Male	158 (27.4)	0.15 (0.09)	
Female	419 (72.6)	0.16 (0.09)	
Age (y)			<0.001
60–69	21 (3.6)	0.14 (0.09)	
70–79	196 (34.0)	0.14 (0.09)	
80–89	292 (50.6)	0.17 (0.09)	
≥90	68 (11.8)	0.18 (0.08)	
Ethnicity			0.86
NZ European	410 (71.1)	0.16 (0.09)	
Other European	147 (25.5)	0.16 (0.09)	
Other Ethnicity	20 (3.4)	0.15 (0.08)	
Marital Status			<0.001
Other	330 (57.2)	0.18 (0.08)	
Married/Civil Union/Defacto	247 (42.8)	0.14 (0.09)	
Living arrangement			<0.001
Alone	352 (61.0)	0.18 (0.08)	
Other	225 (39.0)	0.13 (0.09)	
NZ deprivation index, n (%)			0.01
1–5	442 (76.6)	0.15 (0.08)	
6–10	135 (23.4)	0.18 (0.10)	
Treatment group in RCT			<0.001
MDT intervention	199 (34.4)	0.19 (0.08)	
Usual care/Not enrolled RCT	379 (65.6)	0.14 (0.09)	

Notes, NZ deprivation index: 1 represents the least deprived area, 10 representing the most deprived; One way analysis of variance was used to examine differences in mean frailty index (as continous variable) by characteristics of residents.

**Fig 2:**
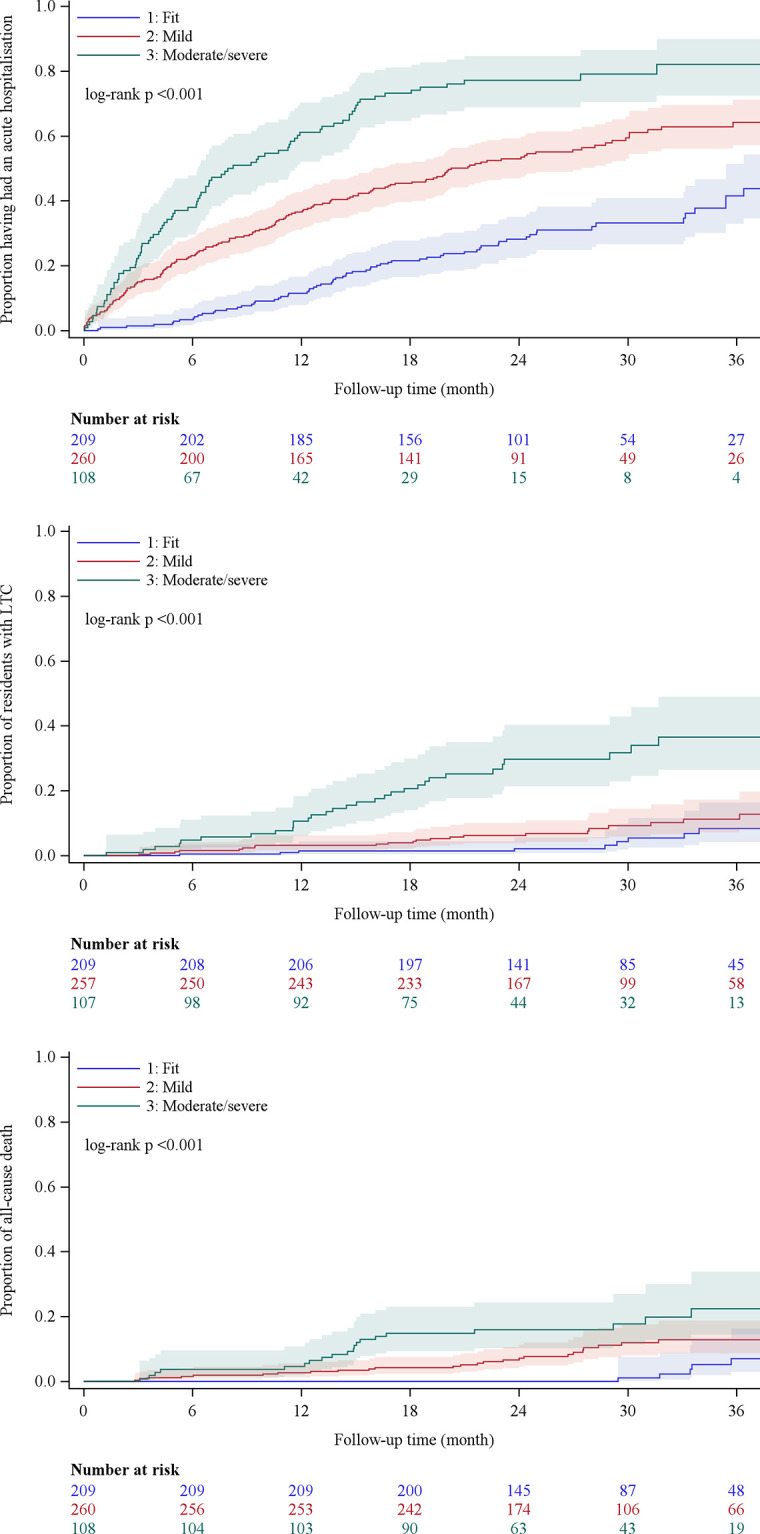
Proportion of residents with a healthcare outcome during follow-up by frailty categories (A) acute hospitalization, (B) LTC, (C) death. Notes, LTC long term care.

Mean follow-up was 2.5 years from baseline assessment. Fig 2 shows the proportion of residents with each outcome during study follow-up by frailty group and the residents at risk over time. There were significant differences by frailty groups in the time to first acute hospitalization (p<0.001), LTC (p<0.001) and death (p<0.001).

Table 2 shows risk of health outcomes by frailty group in unadjusted and adjusted models.

**Table 2:** Proportion of residents having a healthcare outcome during follow-up and hazard ratios by frailty categories.

Outcome	Frailty index, Hazard ratio (95%CI), p			P value for group difference
	Fit	Mild	Moderate or severe	
**Acute hospitalization (n = 577)**	**(n = 209)**	**(n = 260)**	**(n = 108)**	
n (%)	69 (33.0)	152 (58.5)	85 (78.7)	<0.001
Unadjusted model	1.00	2.28 (1.73, 3.00), <0.001	4.51 (3.28, 6.21), <0.001	<0.001
Adjusted model*	1.00	1.88 (1.41, 2.51), <0.001	3.52 (2.53, 4.90), <0.001	<0.001
**LTC (n = 573)** ^†^	**(n = 209)**	**(n = 257)**	**(n = 107)**	
n (%)	9 (4.3)	24 (9.3)	31 (29.0)	<0.001
Unadjusted model	1.00	2.28 (1.06, 4.89), 0.03	8.81 (4.18, 18.60), <0.001	<0.001
Adjusted model*	1.00	1.45 (0.66, 3.21), 0.35	5.60 (2.47, 12.72), <0.001	<0.001
**Death (n = 577)**	**(n = 209)**	**(n = 260)**	**(n = 108)**	
n (%)	5 (2.4)	26 (10.0)	20 (18.5)	<0.001
Unadjusted model	1.00	4.26 (1.65, 11.01), 0.003	5.54 (3.25, 22.46), <0.001	<0.001
Adjusted model*	1.00	2.70 (0.94, 7.74), 0.07	5.06 (1.71, 15.02), 0.003	0.007

Notes

*, adjusted for age at interRAI assessment, gender, ethnicity, marital status and NZ deprivation index, acute hospitalization at baseline (1 year prior interRAI assessment), MDT intervention (as a time-dependent variable); marital status were highly associated with living arrangement (Spearman correlation coefficient = 0.9), so only marital status was adjusted in the multivariable analysis

^†^, 4 residents who received long-term care at the time of interRAI assessment were excluded. CI confidence interval, MDT multidisciplinary team, LTC long term care.

The excess risk for acute hospitalization was significant in mildly frail (adjusted HR = 1.88, 95%CI = 1.41–2.51, p<0.001) and moderate-severely frail (adjusted HR = 3.52, 95%CI = 2.53–4.90, p<0.001) group. For LTC entry the excess risk was significant in both the mild and moderate-severely frail categories in the unadjusted results, but only for moderate-severely frail in adjusted modelling (adjusted HR = 5.60, 95%CI = 2.47–12.72, p<0.001). For mortality, both mild and moderate-severe categories showed significant risk in unadjusted results, but significance persisted only for moderate-severely frail in adjusted modelling (adjusted HR = 5.06, 95%CI = 1.71–15.02, p = 0.003).

Table 3 demonstrates discrimination and overall performance for FI groups for each outcome (S1 Table illustrates this for continuous FI).

**Table 3:** Discrimination and overall performance of frailty index categories for predicting healthcare outcomes.

Outcome	C statistic	Nagelkerke’s R^2^
Acute hospitalization	0.66	0.14
LTC*	0.73	0.07
Death	0.72	0.04

Notes, Harrell’s c statistic was reported; Nagelkerke’s R^2^ statistic which was based on the likelihood-ratio statistic was reported

*, 4 residents who received long-term care at the time of interRAI assessment were excluded. LTC long term care. Nagelkerke’s R^2^ indicates overall performance of FI itself for predicting healthcare outcomes.

Harrell’s concordance (c) statistic indicates the FI discriminative ability.

For acute hospitalizations, LTC entry and mortality, estimates of C-statistic were 0.66, 0.73 and 0.72, and Nagelkerke’s R^2^ were 0.14, 0.07 and 0.04, respectively. The FI demonstrated moderate discrimination for the outcomes of acute hospitalization, LTC and mortality [23]. However, the overall performance (explained variation) of FI itself for predicting the above three outcomes are low, indicating that the FI itself did not explain much variability in those outcomes.

## Discussion

NZ was the first country to mandate the use of interRAI for initiating access to community and residential government-provided supports. However, interRAI tools are also frequently used internationally. Our study suggests robust predictive validity of interRAI-CHA FI for healthcare outcomes (acute hospitalization, LTC and mortality). To the best of our knowledge, this is the first report demonstrating an interRAI-CHA derived FI is independently associated with healthcare outcomes, and has reasonable discriminations for those outcomes. Additionally this is the first exploring frailty measured in this way in RV residents.

The utility of interRAI tools for FI development is attractive, and several other interRAI FIs have been successfully developed, including the ED contact assessment [24], Acute Care (AC) [25, 26] Home Care (HC) [27–29] and Post Acute Care (PAC) [30]. The advantages of interRAI based tools are the comprehensive and multi-dimensional items that are included, consistency of item assessment by trained personnel, and potential for comparison across international populations. The briefer contact assessment and longer HC can also be used for assessing community-dwelling older adults. The CHA has advantages over these tools with its intermediate number of items, and allowing for FS completion when triggered in subjects requiring more detailed assessment—a time advantage for individual assessors and participants.

An interRAI-HC FI tool has previously explored frailty in NZ [27, 28] and elsewhere [29, 31–33] in the community setting and assisted living (AL) residents. Subjects in all of these studies were all requiring funded assistance [27, 29, 31, 32], or had intellectual or developmental disabilities [33], and therefore were not representative of all community dwellers. Mean FI in these studies ranged from 0.19 to 0.27 [27, 29, 33], and in those utilizing frailty cut-offs higher values were used (≥0.21–0.3 pre-frail, >0.3 frail) than in our study [29, 31–33]. In those reporting adverse outcomes, higher FI scores were associated with hospitalization, mortality and LTC [27, 28, 31] with Hogan et al. reporting risk ratios (RR) for hospitalizations of 1.37 (95%CI 1.13–1.66) and 1.28 (95%CI 1.04–1.57) for prefrail and frail respectively, in an AL population. In comparison, FI in UK community dwellers reported one-year adjusted HRs for hospitalization of 1.93 (95%CI 1.86–2.01), 3.04 (95%CI 2.90–3.19) and 4.73 (95%CI 4.43–5.06) for mild, moderate and severe frailty respectively [18], similar to our results. A meta-analysis of frailty measured by different tools reported odds ratios (OR) of 1.82 (95%CI 1.53–2.15), HR/RR 1.18 (95%CI 1.10–1.28) for hospitalization, OR 2.34 (95%CI 1.77–3.09), HR/RR 1.83 (95%CI1.68–1.98) for mortality and OR 1.69 (95%CI 1.02–2.81), HR/RR 1.65 (95%CI 1.48–1.84) for LTC [34]. A more recent systematic review and meta-analysis of frailty and LTC risk reported pooled odds ratios of 3.26 (95%CI 1.21–8.78) and 5.58 (95%CI 2.94–10.60) for prefrail and frail community dwellers, more in keeping with risks reported in our study [35].

Our study specifically addresses frailty within the RV setting, not otherwise explored to date in this way, and with many residents not requiring formal provision of cares. HenceenceHence, our findings are more likely closer to frailty in the wider NZ European community-dwelling older adult population.

We found after adjusting for confounders, FI predicted acute hospitalization in all levels of frailty and increased risk of LTC entry and mortality persisted in those moderately-severely frail, consistent with literature in community-dwelling older adults [34–36]. This identification could allow healthcare providers to focus attention on potential reversible factors, which may ameliorate frailty (or its consequences), such as falls prevention, chronic disease management, appropriate medications, nutrition, exercise and advance care planning facilitation. In the unadjusted analysis, all levels of frailty predicted LTC and mortality risk, however this was lost in adjusted data for those with mild frailty, suggesting other factors influenced these outcomes in these residents. This also likely reflects the low numbers of LTC entry and mortality outcomes in this population over the follow up period, and larger studies over a longer follow up maybe needed to better explore these associations. Further cohort studies are required for these outcomes.

Limitations: Due to issues accessing residents in some RVs [12], most participants were volunteers. Furthermore, our data only captures those who had publicly funded entry into LTC. Additionally, due to NZ legislation and ethical guidelines we could not include residents who had significant cognitive impairment, even with a legal representative present. NZ’s RV community has very little ethnic diversity, and does not capture the significant number of Māori, Pasifika, Asian or other older adult groups that reside in Auckland. As above, we are aware of other studies using different cut-offs for classification of frailty to those used here [29–32]. These involve populations with greater functional needs than our RV population, and we chose to use cut-offs described by Clegg et al. [18]. Utilising higher cut off points in our study would have potentially meant those on the lower end of the frailty spectrum with increased risk of hospitalizations may not have been identified. Similar to other FI studies, the overall predictive *performance* (explained variation) of FI for healthcare outcomes was low in this study as indicated by estimates of Nagelkerke’s R^2^, meaning FI itself does not explain much variability within these outcomes, the predictive risk of an individual resident may not be precise (more variation). However, our primary objective was interRAI-CHA FI development and predictive validity assessment, which was informed by, and consistent with, published literature [18].

## Conclusions and implications

The FI derived from the versatile interRAI-CHA tool suggests those with increased frailty are more likely to suffer adverse health outcomes after adjusting for confounding factors. Given many frail older adults see RV living as a way to live supported in the community without the need for LTC [5], it is imperative that we understand this population in terms of frailty and whether their health, social and functional needs are met within RV/CCRC models. Such predictive validation allows us to now further study frailty within the context of retirement communities and facilities—currently an unexplored area. If our findings are replicated by further large-scale cohort studies, use of interRAI derived FIs embedded within the health systems in which interRAI is used would allow rapid identification and intervention of at risk individuals. In NZ interRAI is currently only mandated for assessing community dwellers for community home-based assistance or for government funded admission to LTC. Our results, however, demonstrate that risk of adverse outcomes goes beyond just those receiving such supports. RVs are perhaps in a good position to utilize interRAI-CHA FIs to identify potential residents at risk and may be well-positioned to offer frailty prevention programmes, given many, but not all, already offer programmes for physical activity, on-site primary care clinics, or community rooms ideal for health education programmes [2]. RV-based frailty programmes sitting within residents’ communities could potentially enhance uptake, acceptability and continuity of such interventions.

## Supporting information

S1 Table Discrimination and overall performance of continuous frailty index for predicting the healthcare outcomes. (DOCX)
