# Supplementary material for: An interRAI derived frailty index predicts acute hospitalizations in older adults residing in retirement villages: A prospective cohort study
Source: PLoS One. 2022 Mar 2;17(3):e0264715. doi: 10.1371/journal.pone.0264715 (PMC8890727; doi:10.1371/journal.pone.0264715)
Supplement: S1 Table — (DOCX) [file pone.0264715.s001.docx]

**S1 Table: Discrimination and overall performance of continuous frailty index for predicting the healthcare outcomes.**

| **Outcome** | **C statistic** | **R^2^** |
| --- | --- | --- |
| Acute hospitalisation | 0.68 | 0.14 |
| LTC^1^ | 0.75 | 0.07 |
| Death | 0.74 | 0.04 |

Notes, Harrell’s c statistic was reported; Nagelkerke’s R^2^ statistic which was based on the likelihood-ratio statistic was reported; ^1^, 4 residents who were received long-term care at the time of interRAI assessment were excluded.
